# Distinct Inflammatory Mediator Patterns Characterize Infectious and Sterile Systemic Inflammation in Febrile Neutropenic Hematology Patients

**DOI:** 10.1371/journal.pone.0092319

**Published:** 2014-03-18

**Authors:** Christine Wennerås, Lars Hagberg, Rune Andersson, Lars Hynsjö, Anders Lindahl, Marcin Okroj, Anna M. Blom, Peter Johansson, Björn Andreasson, Johan Gottfries, Agnes E. Wold

**Affiliations:** 1 Department of Hematology and Coagulation, Sahlgrenska Academy, University of Gothenburg, Göteborg, Sweden; 2 Department of Infectious Diseases, Sahlgrenska Academy, University of Gothenburg, Göteborg, Sweden; 3 Department of Clinical Chemistry, Sahlgrenska University Hospital, Göteborg, Sweden; 4 Department of Laboratory Medicine, Lund University, Malmö, Sweden; 5 Department of Internal Medicine, Uddevalla Hospital, Uddevalla, Sweden; 6 Department of Chemistry and Molecular Biology, University of Gothenburg, Göteborg, Sweden; The Hospital for Sick Children and The University of Toronto, Canada

## Abstract

**Background:**

Invasive infections and sterile tissue damage can both give rise to systemic inflammation with fever and production of inflammatory mediators. This makes it difficult to diagnose infections in patients who are already inflamed, e.g. due to cell and tissue damage. For example, fever in patients with hematological malignancies may depend on infection, lysis of malignant cells, and/or chemotherapy-induced mucosal damage. We hypothesized that it would be possible to distinguish patterns of inflammatory mediators characterizing infectious and non-infectious causes of inflammation, respectively. Analysis of a broad range of parameters using a multivariate method of pattern recognition was done for this purpose.

**Methods:**

In this prospective study, febrile (>38°C) neutropenic patients (n = 42) with hematologic malignancies were classified as having or not having a microbiologically defined infection by an infectious disease specialist. In parallel, blood was analyzed for 116 biomarkers, and 23 clinical variables were recorded for each patient. Using O-PLS (orthogonal projection to latent structures), a model was constructed based on these 139 variables that could separate the infected from the non-infected patients. Non-discriminatory variables were discarded until a final model was reached. Finally, the capacity of this model to accurately classify a validation set of febrile neutropenic patients (n = 10) as infected or non-infected was tested.

**Results:**

A model that could segregate infected from non-infected patients was achieved based on discrete differences in the levels of 40 variables. These variables included acute phase proteins, cytokines, measures of coagulation, metabolism, organ stress and iron turn-over. The model correctly identified the infectious status of nine out of ten subsequently recruited febrile neutropenic hematology patients.

**Conclusions:**

It is possible to separate patients with infectious inflammation from those with sterile inflammation based on inflammatory mediator patterns. This strategy could be developed into a decision-making tool for diverse clinical applications.

## Introduction

Systemic inflammation is a complex reaction of the body to external and internal threats. It encompasses fever, activation of white blood cells and of the complement and coagulation systems, production of acute phase proteins by the liver, and altered metabolism and function of many organ systems. The inflammatory cascade is triggered by “danger signals” [1]. These signals may originate from microbes, ”pathogen-associated molecular patterns” (PAMPs) [2], e.g. lipopolysaccharide, peptidoglycan, β-glucan, and microbial DNA. Inflammation can also be triggered by substances leaking out of our own injured tissues, i.e. ”damage-associated molecular patterns” (DAMPs), such as ATP, uric acid and mitochondrial *N*-formylated peptides [3]. The inflammatory response is fairly stereotypic, regardless of the initiating cause. Thus, severe infections and sterile tissue injury arising from trauma or tumor cell decay, are all conditions that are associated with fever and elevated levels of acute phase proteins in the blood.

Patients with hematologic malignancies are at high risk of contracting invasive infections for several reasons. Chemotherapy damages the mucosal barriers, which together with central venous lines used to administer chemotherapy, facilitate microbial invasion. Neutropenia frequently develops both due to the toxic effects of chemotherapy and the expansion of malignant cells, which hinder the growth of hematopoietic cells in the bone marrow. Neutrophils are central in the defense against bacteria and fungi, consequently, neutropenic patients are exceedingly susceptible to these infections [4].

Rapid diagnosis of infections is essential since delay of targeted antibiotic treatment is associated with high mortality rates in neutropenic hematology patients [5], [6], [7], [8]. Secondly, chemotherapy and other immunosuppressive interventions must be postponed in patients with severe infections. However, the diagnosis of infections in this patient group is difficult since they are already inflamed due to intermittent exposure to DAMP signals released from cells damaged by treatment with cytotoxic drugs. In fact, patients with hematologic malignancies undergoing intensive chemotherapy often display several criteria of the systemic inflammatory reaction syndrome (SIRS) [9], such as fever, increased heart rate, and reduced white blood cell counts in the absence of invasive microbial infection. Estimates indicate that every other febrile episode afflicting these patients is due to sterile inflammation, e.g. chemotherapy-induced cell lysis, drug reactions, decaying malignant cells [10]. It is equally important to exclude ongoing invasive infection in hematology patients with DAMP-driven inflammation, because prolonged interruptions of chemotherapy may compromise the chance of curing the underlying malignancy.

The search for a single or handful of laboratory parameter(s) able to distinguish between the inflammatory response triggered by a severe infection, as opposed to one evoked by sterile cell decay, has hitherto been futile [11]. The aim of this study was to identify combinations of clinical, physiological and laboratory parameters that could separate infectious from non-infectious causes of fever in neutropenic hematology patients. Two assumptions were made: 1) Inflammation elicited by microbes will differ from that evoked by sterile tissue damage by discrete alterations in the levels of many inflammatory parameters. 2) In addition to classical inflammatory mediators, parameters reflecting metabolism and organ function will provide valuable information regarding the nature and cause of systemic inflammation.

In order to identify relevant parameter combinations for segregation of infectious from non-infectious inflammation, computational methods able to handle hundreds of parameters are required. “Pattern recognition methods” have been used successfully in proteomics, genomics and metabonomics to analyze large data sets [12]. Here, we employed O-PLS (orthogonal projection onto latent structures) [13], a development of principal component analysis, to search for a specific inflammatory pattern signaling invasive infection in neutropenic hematology patients with fever.

## Methods

### Patients and study design

The clinical characteristics of the 52 patients included in this prospective study are summarized in **Table 1**. The patients were recruited at the Departments of Hematology at Sahlgrenska University Hospital (n = 50) and Uddevalla Hospital (n = 2). Inclusion criteria were neutropenia (<0.5×10^9^/L) and fever (body temperature ≥38.0°C) on the day of inclusion, or the previous day. No restrictions were made regarding the duration of the febrile episode, if patients were fasting or not, if blood was drawn from indwelling catheters or other devices. All study persons gave informed written consent and the study was approved by the Regional Ethical Review Board of Göteborg.

**Table 1:** Clinical characteristics of hematology patients.

	Prediction set (n = 42)		Validation set (n = 10)	
	Number	Percent	Number	Percent
**Demographic**				
Female sex	17	40	3	30
Age, median (min-max)	56 (25–75)		60 (19–74)	
No of fever days (min-max)	2 (1–20)		2 (1–14)	
				
**Hematologic disease**				
Acute myeloid leukemia	15	36	4	40
Malignant lymphoma	11	26	2	20
Multiple myeloma/plasma cell leukemia	8	19	2	20
Acute lymphoblastic leukemia	4	9.5	1	10
Chronic lymphocytic leukemia	3	7.1	0	0
Other disorders	1	2.4	1	10
				
**Hematopoietic stem cell transplantation**				
Autologous	4	9.5	1	10
Allogeneic	6	14	1	10
				
**Immunosuppressive treatment**				
Chemotherapy, ongoing	2	4.8	1	10
Corticosteroids, systemic	8	19	2	20
Cyclosporin A, ongoing	5	12	1	10
Immunosuppression, previous 3 months	42	100	10	100
				
**Supportive treatment**				
Granulocyte-colony stimulating factor	7	17	4	40
Total parenteral nutrition	13	31	3	30
				
**Ongoing antibiotics**				
Antibacterial	36	86	10	100
Antifungal	24	57	5	50
Antiviral	38	90	9	90

The patients were defined as having or not having a microbiologically defined infection. In parallel, a broad range of biochemical parameters were measured. The first set of patients (n = 42), the prediction set, served to construct a model for distinguishing infected from non-infected patients, whereas the second set constituted the validation set (n = 10), for testing the predictive capacity of the model.

### Identification of microbiologically defined infections

The patients were defined as being infected or not based on extensive microbiological screening. Blood cultures were performed and cytomegalovirus (CMV) genome equivalents were determined in serum by quantitative PCR. Pan-bacterial (16SrRNA) PCR was done to detect non-cultivatable bacteria [14], and levels of serum-galactomannan and β-1-3-glucan were assessed to detect fungal antigens. Depending on symptoms and clinical signs, additional cultures were done, e.g. of urine, bronchoalveolar lavage fluid, cerebrospinal fluid, throat swabs, wounds, and central venous catheter insertion sites. Patients were screened for HIV and hepatitis B and C viruses. All clinical microbiological analyses were performed at the Dept. of Clinical Microbiology, Sahlgrenska University Hospital, using validated methods; the laboratory is accredited by the Swedish Board for Accreditation and Conformity Assessment (SWEDAC) in accordance with International Standard ISO 15189:2007. CT scans of the thorax, abdomen and brain, chest X-rays and ultrasound scans were performed guided by the clinical picture.

Patients were classified as having a “microbiologically defined infection” or not, in accordance with the guidelines provided by the Immunocompromised Host Society [15]. For fungal infections, the latest EORTC/MSG criteria for definition of proven or probable invasive fungal disease were used [16]. The patients in the prediction set (n = 42) were categorized as having or not having a microbiologically defined infection by an experienced infectious disease specialist (LHa), who had neither seen the patients nor the results of the biochemical analyses. The validation set of patients (n = 10) was categorized in the same manner by two infectious disease specialists (LHa and RA), independent of one another. The following data were presented to the evaluators: 1) clinical signs and symptoms manifested by the patients, 2) microbial findings, and 3) imaging data.

### Clinical parameters

All patients underwent a brief physical exam and interview to monitor clinical signs and anamnestic clinical data. Clinical data were also retrieved from patient charts. The collected clinical data are shown in **Table 2**.

**Table 2:** Clinical, physiological and blood parameters measured in patients included in the prediction set.

Category	Method	n	Analytes
Clinical	Anamnesis	10	Age, sex, length, weight, smoking, previous autologous or allogeneic hematopoietic stem cell transplantation, hypogammaglobulinemia, number of fever days prior to inclusion, presence of chills
Current medication	Anamnesis	3	Corticosteroids, cytarabine, cyclosporin A
Physiology	Examination	5	Body temperature, systolic and diastolic blood pressure, heart rate, peripheral oxygen saturation
Sampling	Anamnesis	5	Fasting at the time of sampling, peripheral vein catheter, central venous catheter, port-a-catheter, sampling via peripheral vein
Blood cells and related variables	Blood analysis	14	White blood cell counts (WBC), monocytes, neutrophils, basophils, eosinophils, lymphocytes, platelet counts (PC), erythrocyte particle counts (EPC), erythrocyte mean corpuscular volume (E-MCV), erythrocyte volume fraction (EVF), erythrocyte mean corpuscular hemoglobin concentration (MCH, MCHC), reticulocytes, hemoglobin
Complement	Plasma analysis	5	Complement factor 3 (C3), C4, C4-binding protein (C4BP)-β, C4BP-β, total complement complex (TCC)
Coagulation and fibrinolysis	Plasma analysis	17	Activated partial thromboplastin time (APTT), prothrombin time (measured as international normalized ratio, INR), coagulation factors II (prothrombin), V, VII, VIII, IX, X, XI, XII, von Willebrand factor, fibrinogen, anti-thrombin, D-dimers, plasminogen activator inhibitor-1 (PAI-1), protein C, protein S
Cytokines	Serum analysis	8	Interferon-γ (IFN-γ, Interleukin-1 (IL-1), IL-6, IL-8, IL-10, IL-17, tumor necrosis factor (TNF), lymphotoxin (LT)
Acute phase proteins	Serum analysis	16	C-reactive protein (CRP), haptoglobin, hemopexin, orosomucoid, serum amyloid A (SAA), α1-antitrypsin, α2-macroglobulin, ceruloplasmin, ferritin, hepcidin, pro-hepcidin, soluble transferrin receptor, total iron binding capacity (TIBC), transferrin, albumin, procalcitonin
Acute phase response	Blood analysis	1	Erythrocyte sedimentation rate (SR)
Kidney function and electrolytes	Serum analysis	13	Sodium (Na), potassium (K), Calcium (Ca), chloride (Cl), magnesium (Mg), phosphate (P), iron, urea, creatinine, cystatin C, NT-pro-brain natriuretic peptide (NT-pro-BNP), erythropoietin (EPO), renin
Liver	Serum analysis	6	Aspartate aminotransferase (AST), alanine aminotransferase (ALT), alkaline phosphatase (ALP), bilirubin conjugated, bilirubin total, γ-glutamyl transferase (γGT)
Organ stress and cell decay	Serum analysis	13	Creatine kinase (CK), creatine kinase-muscle brain (CK-MB), troponin-T, lactate dehydrogenase (LDH), myoglobin, pancreatic amylase, total amylase, prostate specific antigen (PSA), free PSA, high mobility group box protein-1 (HMBG1), α-fetoprotein (AFP), urate, lactoferrin (plasma)
Metabolism	Serum analysis	8	High density lipoprotein (HDL), low density lipoprotein (LDL), cholesterol, triglycerides, protein, leptin, adiponectin, glycosylated hemoglobin (HbA1c)
Metabolism	Plasma analysis	1	Glucose
Hormones	Serum analysis	10	Cortisol, T4 (tetraiodo-thyronine, thyroxine), free T4, T3 (triiodo-thyronine), free T3, thyroxine-binding globulin (TBG), thyroid-stimulating hormone (TSH), insulin-like growth factor-1 (IGF-1), estradiol, testosterone
Immunoglobulins	Serum analysis	4	Immunoglobulin A (IgA), IgE, IgG, IgM
Total number		139	

### Biochemical parameters

Venous blood (40 ml) was collected into EDTA-, citrate-, heparin-, SST- or un-treated test tubes. Routine clinical chemistry analyses were immediately performed on fresh blood. **Aliquots of plasma and serum were stored at -80°C for later analyses.**

A total of 116 biochemical and immunological analyses were performed on blood (B), serum (S) or blood plasma (P), **Table 2**. Most analyses were done at the Department of Clinical Chemistry, Sahlgrenska University Hospital, using automated procedures and accredited methods. Quantitation of citrulline in heparinized plasma was based on ion exchange chromatograpy with ninhydrin staining. Terminal complement complexes and C4-binding protein were assessed by ELISA [17], at the Dept. of Laboratory Medicine, Malmö, Sweden. Serum levels of the cytokines Interleukin-1β (IL-1), IL-6, IL-8, IL-10, tumor necrosis factor and interferon-γ were analyzed using the Cytokine Bead Array Kit (BD Biosciences), or ELISA (IL-17-α, R&D Systems; TNF-β; eBioScience). HMBG1 was determined by EIA (IBL Gesellschaft). The limits of detection were: IL-1 2.3 pg/ml, IL-6 1.6 pg/ml, IL-8 1.2 pg/ml, IL-10 0.13 pg/ml, TNF 0.7 pg/ml, IFN-γ 1.8 pg/ml, IL-17 and HMGB1 1.0 ng/ml.

### Statistics

The multivariate method Orthogonal Partial Least Squares Projections to Latent Structures (O-PLS) was employed using SIMCA-P 12.0 software (Umetrics, Sweden) for construction of a model to predict infection (Y-variable) based on a matrix of clinical and biochemical parameters (X-variables) [13], [18]. In O-PLS, each model is defined by R^2^Y, which estimates the fraction of the variance in Y explained by the X-matrix, and Q^2^Y, which describes the validity of the model. The latter is determined via cross-validation, i.e. a “leave data out” procedure, where an equal number of objects (i.e. patients) are removed, and the capacity of the remaining data to predict Y is assessed. Jack-knifing was used to estimate the uncertainty of calculated scores and loadings, indicated by confidence intervals [19]. Prior to all calculations, X-variables with >10-fold distribution were log-transformed using the SIMCA transformation tool. Mean-centering and unit variance scaling were implemented to give all variables an equal chance of providing model leverage independently of data scale and distribution. To remove unnecessary variables, the “Variable importance” module of the SIMCA software was used. The prediction module was employed for validation of the model using the validation set of patients. Non-parametric, two-tailed Mann-Whitney U test was used for univariate analyses (GraphPad Prism 5.0).

## Results

### It is not possible to separate infected from non-infected febrile neutropenic patients based on analyses of single inflammation parameters

Each of the patients of the prediction set (n = 42) was classified as having or not having a microbiologically defined infection by an experienced infectious disease specialist. A third of the patients (13/42) were classified as infected. The majority (11/13) had bacterial sepsis (4 cases of α-streptococci, 3 enterococci, 1 *Bacillus* sp., 1 *Gemella* sp., 1 *Pseudomonas aeruginosa*, 1 *E. coli*). In addition, one case each of invasive fungal disease (*Pneumocystis jiroveci* pneumonia) and viral reactivation (CMV) was seen.

**Figure 1:**
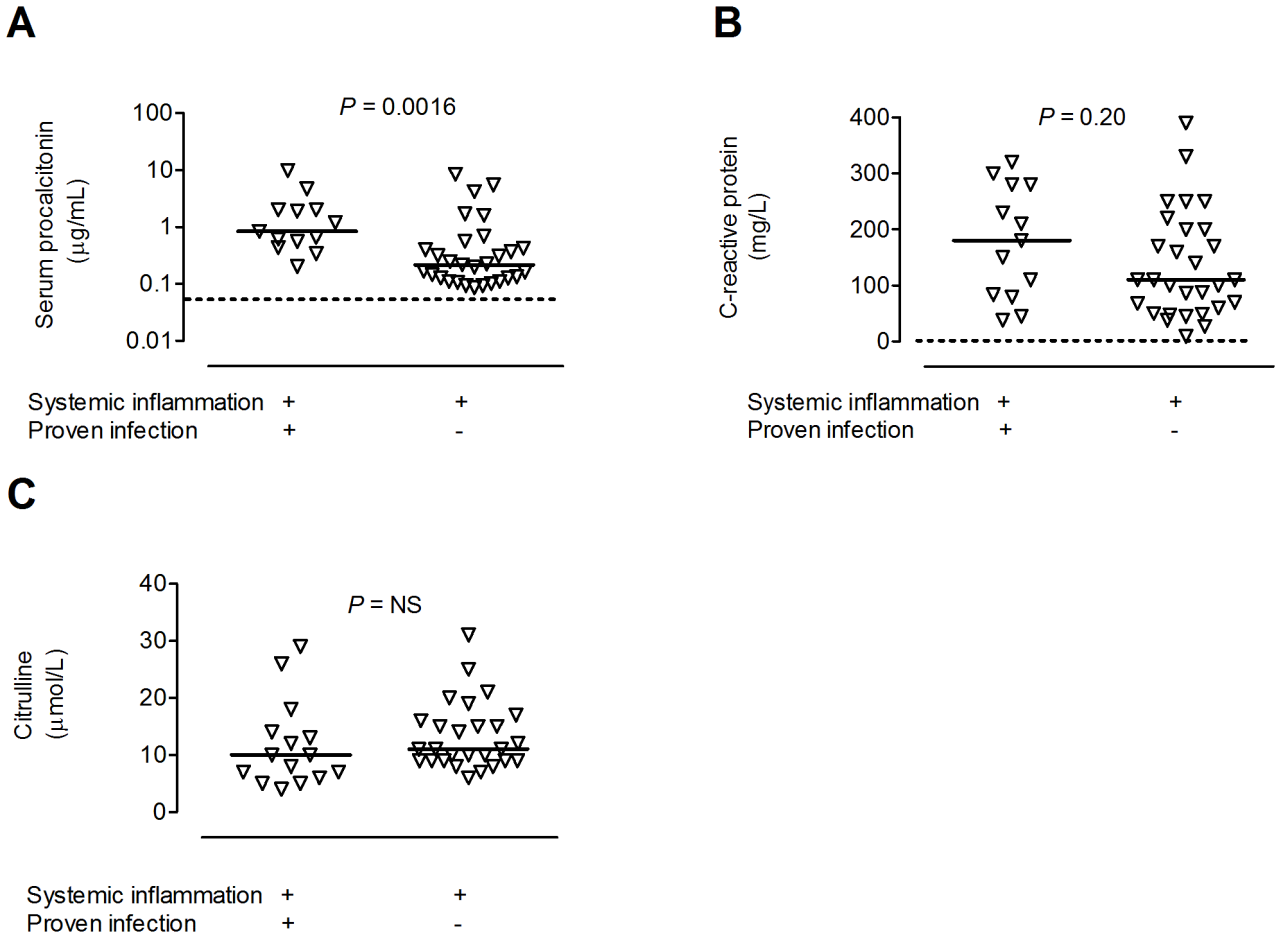
Elevated and overlapping levels of markers of inflammation and tissue damage in febrile neutropenic hematology patients with or without infection. Serum levels of (A) procalcitonin, (B) C-reactive protein, and plasma levels of (C) citrulline in patients with (n = 13) and without proven infection (n = 29). Each symbol denotes one patient and the horizontal line indicates the median. Dashed line shows upper limit of normal reference interval for procalcitonin and CRP. Normal level of citrulline in healthy individuals is >20 μmol/L [21], [41]. Statistical significance was determined by the Mann-Whitney test.

Both patients with and without proven infections were severely inflamed, i.e., all patients had serum procalcitonin and C-reactive protein levels above the normal range, and there was considerable overlap between the groups (**Figure 1A and B**). Moreover, plasma levels of citrulline were in the same range in the infected as in the non-infected patients (**Figure 1C**). This amino acid is produced by enterocytes and lowered levels are associated with mucocitis and other forms of mucosal/intestinal inflammation.

### Infected and non-infected systemically inflamed patients can be separated based on pattern recognition modelling

**Figure 2:**
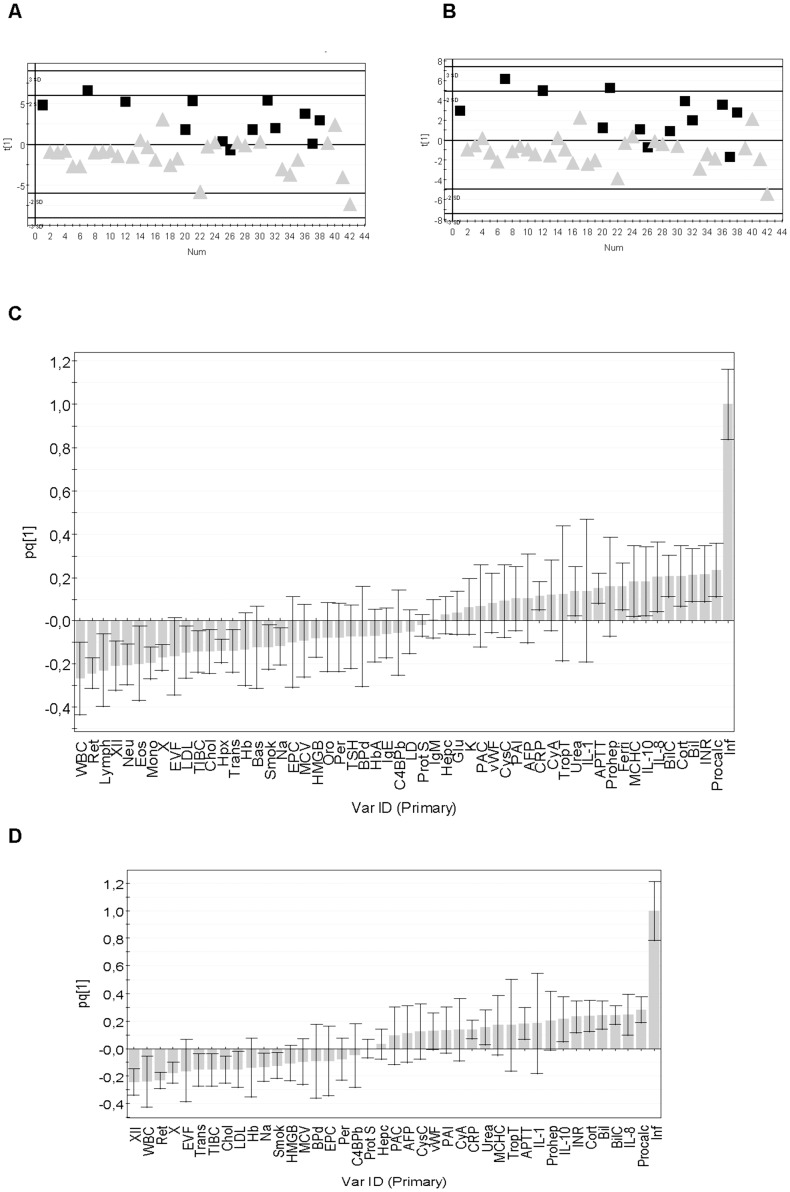
Pattern recognition models can separate infected from non-infected hematology patients with neutropenic fever. (A) O-PLS scatter plot showing how the patients (n = 42) separate in a model based on 139 parameters; black symbols denote patients with proven infection, grey symbols those without proven infection. “Num” on the X-axis indicates arbitrary number assigned to each patient. The Y-axis measure, t [1], indicates the probability of being infected according to the model. The model had an explanatory power of 47% (R^2^Y = 0.47) but poor cross-validation (Q^2^Y = −0.0082). (B) Scatter plot of a model based on 55 of the original 139 variables; variables of low importance (VIP values <1.0) were removed. R^2^Y = 0.50, Q^2^Y = 0.32. (C) Column loading plot showing how the 55 variables contribute to the model shown in B. Variables positively associated with infection (Inf) are positioned above the line in the right half of the diagram, those that correlate negatively with infection are positioned below the X-axis. The larger the bar, the larger is the contribution made by the variable to the model. The smaller the confidence interval (error bar), the more certain is the contribution of the variable in question. (D) Loading plot of the model based on 40 variables. R^2^Y = 0.47, Q^2^Y = 0.30.

The multivariate pattern recognition method O-PLS was used to construct a model that could separate the patients with a microbiologically defined infection (Y = 1) from those without a microbiologically defined infection (Y = 0) based on a broad range of biochemical (n = 116) and clinical parameters (n = 23), **Table 2**. As shown in **Figure 2A**, the infected patients could be separated from the non-infected ones using a model based on the 139 variables. The contribution of each variable to the model was determined using the “Variable importance” (VIP) approach. Serum procalcitonin was the variable that contributed most to the model, i.e., had the highest VIP-value (**Table 3**). Other important variables included the routine clinical analytes S-urea, S-bilirubin, B-hemoglobin, white and red blood cell counts, as well as interleukins-8 and -10, and proteins involved in coagulation and fibrinolysis, e.g. factor XII, plasminogen-activator inhibitor-1 and von Willebrand factor (**Table 3**). Parameters with negligible contribution to the model appear at the bottom of **Table 3**, and encompass many classical measures of inflammation, e.g. acute phase proteins, complement factors, and several cytokines; these parameters were either not affected by inflammation or similarly altered in the infected and non-infected patients. The presence of chills, degree of elevation of body temperature, number of days with fever, heart rate, and peripheral oxygen saturation rate were also of no value for separating infected from non-infected patients (**Table 3**). Current treatment with cyclosporine A was more common in infected than non-infected patients, whereas neither treatment with cytosar (a chemotherapeutic drug frequently associated with fever) or corticosteroids helped to discriminate the infected from the non-infected subjects (**Table 3**).

**Table 3:** Contribution of biochemical and physical variables to a model predicting infection in systemically inflamed neutropenic hematology patients.

Variable			Patient group with highest level of analyte	Included in model	
Name	Short name	Impor-tance^a^		55-variable	40-variable
S-Procalcitonin	Procalc	2.5	Infected^b^	X	X
S-Urea	Urea	2.3	Non-infected^b^	X	X
B-White blood cell count	WBC	2.3	Infected	X	X
S-Interleukin-10	IL-10	2.2	Infected	X	X
S-Bilirubin	Bil	2.0	Infected	X	X
S-Interleukin-8	IL-8	1.9	Infected	X	X
P-Factor XII (Hageman factor)	XII	1.9	Non-infected	X	X
B-Erythrocyte volume fraction	EVF	1.8	Non-infected	X	X
S-Bilirubin (conjugated)	BilC	1.8	Infected	X	X
B-Hemoglobin	Hb	1.7	Non-infected	X	X
Current smoker	Smok	1.7	Non-infected	X	X
S-Cortisol	Cort	1.7	Infected	X	X
B-Lymphocyte count	Lymph	1.6	Non-infected	X	
P-Plasminogen activator inhibitor-1	PAI	1.6	Infected	X	X
P-von Willebrand factor	vWF	1.6	Infected	X	X
S-High mobility group box protein-1	HMGB	1.6	Non-infected	X	X
S-Prohepcidin	Prohep	1.5	Infected	X	X
S-Low-density lipoprotein	LDL	1.5	Non-infected	X	X
B-Neutrophil count	Neu	1.5	Non-infected	X	
S-C4b-binding protein (β-chain-containing isoform)	C4BPb	1.4	Non-infected	X	X
S-Cystatin C	CysC	1.4	Infected	X	X
P-Glucose	Glu	1.4	Infected	X	
P-Protein S	Prot S	1.4	Non-infected	X	X
Cyclosporin A treatment	CyA	1.3	Infected	X	X
S-Hepcidin	Hepc	1.3	Infected	X	X
B-Mean corpuscular hemoglobin concentration	MCHC	1.3	Infected	X	X
B-Eosinophil count	Eos	1.3	Non-infected	X	
S-Alpha fetoprotein	AFP	1.3	Infected	X	X
S-Lactate dehydrogenase	LD	1.3	Non-infected	X	
Blood pressure (diastolic)	BPd	1.3	Non-infected	X	X
S-Cholesterol	Chol	1.3	Non-infected	X	X
B-Reticulocyte count	Ret	1.3	Non-infected	X	X
S-Immunoglobulin E	IgE	1.3	Non-infected	X	
Peripheral vein sampling	Per	1.2	Non-infected	X	X
Port-a-catheter	PAC	1.2	Infected	X	X
S-Interleukin-1	IL-1	1.2	Infected	X	X
B-Mean corpuscular volume	MCV	1.2	Non-infected	X	X
B-International Normalized Ratio ( = prothrombin time)	INR	1.2	Infected	X	X
P-Factor X	X	1.2	Non-infected	X	X
B-Monocyte count	Mono	1.2	Non-infected	X	
S-C-reactive protein	CRP	1.2	Infected	X	X
S-Ferritin	Ferri	1.1	Infected	X	
S-Potassium	K	1.1	Infected	X	
S-Hemopexin	Hpx	1.1	Non-infected	X	
S-Troponin T	TropT	1.1	Infected	X	X
B-Hemoglobin A	HbA	1.1	Non-infected	X	
S-Transferrin	Trans	1.1	Non-infected	X	X
S-Immunoglobulin M	IgM	1.0	Infected	X	
S-Sodium	Na	1.0	Non-infected	X	X
B-Basophil count	Bas	1.0	Non-infected	X	
B-Erythrocyte particle concentration	EPC	1.0	Non-infected	X	X
S-Orosomucoid	Oro	1.0	Non-infected	X	
P-Activated partial thromboplastin time	APTT	1.0	Infected	X	X
S-Thyroid stimulatory hormone	TSH	1.0	Non-infected	X	
S-Total iron binding capacity	TIBC	1.0	Non-infected	X	X
Allogeneic transplant recipient	Allo	0.97			
P-Coagulation factor II (prothrombin)	II	0.96			
Erythrocyte sedimentation rate	SR	0.91			
S-High density lipoprotein	HDL	0.88			
S-Chloride	Cl	0.85			
S-Amylase, pancreatic	AmyP	0.83			
S-Alanine aminotransferase	ALT	0.81			
S-Immunoglobulin G	IgG	0.79			
S-Albumin	Alb	0.79			
B-Platelet count	PC	0.78			
P-Coagulation factor VII	VII	0.72			
S-Testosterone	Test	0.71			
S-Interleukin-6	IL-6	0.70			
S-Triiodothyronine	T3	0.69			
S-Creatinine	Crea	0.68			
S-Leptin	Lep	0.68			
S-Myoglobin	Myo	0.67			
Blood pressure, systolic	BPs	0.67			
S-Tetraiodothyronine	T4	0.67			
S-Tumor necrosis factor	TNF	0.65			
S-Estrogen	Est	0.65			
S-Protein	Prot	0.64			
Patient fasting prior to blood sampling	Fast	0.63			
S-Creatine kinase, muscle brain	CKM	0.63			
S-Transferrin saturation	TransS	0.60			
P-Factor XI	XI	0.60			
S-Triglycerides	TG	0.60			
S-α2-macroglobulin	a2M	0.59			
S-Aspartate aminotransferase	AST	0.59			
S-Soluble transferrin receptor	TfR	0.57			
Body length	Leng	0.56			
P-Protein C	ProtC	0.55			
Cytarabine treatment, current	Cytar	0.53			
S-Iron	Fe	0.52			
No of fever days prior to inclusion	Fday	0.52			
S-Urate	Urate	0.52			
S-Insulin growth factor-1	IGF-1	0.51			
Peripheral vein catheter	PVC	0.50			
S-Ceruloplasmin	Cer	0.49			
Body weight	Wt	0.48			
S-Phosphate	P	0.48			
S-Haptoglobin	Hap	0.47			
P-Factor VIII	VIII	0.47			
S-Immunoglobulin A	IgA	0.47			
Autologous transplant recipient	Auto	0.47			
S-Alkaline phosphatase	ALP	0.46			
Heart rate	HR	0.45			
B-Erythrocyte mean corpuscular hemoglobin	MCH	0.44			
P-Coagulation factor V	V	0.39			
S-Tetratiodothyronine, free	T4f	0.39			
S-Adiponectin	Adip	0.38			
S-Prostate specific antigen, free	PSAf	0.37			
S-γ-Glutamyl transferase	gGT	0.32			
P-Dimerized plasmin fragment D	Dim	0.32			
S-Thyroxin-binding globulin	TBG	0.32			
Age	Age	0.28			
P-Erythropoietin	EPO	0.27			
Central venous catheter	CVC	0.27			
S-Interferon-γ	IFNg	0.27			
S-Triiodothyronine, free	T3f	0.26			
Hypogammaglobulinemia	Hypog	0.26			
S-Calcium	Ca	0.25			
S-Amyloid A	SAA	0.24			
S-Fibrinogen	Fibrino	0.24			
Temperature	Temp	0.22			
Presence of chills	Chills	0.20			
Peripheral oxygen saturation	POX	0.19			
S-Interleukin-17	IL-17	0.19			
S-C4b-binding protein-α chain	C4BPa	0.18			
S-Complement factor 3	C3	0.17			
Sex	Sex	0.16			
S-Lactoferrin	Lact	0.16			
P-anti-thrombin	a-thrombin	0.14			
S-Amylase, total	AmyT	0.14			
S-Creatine kinase	CK	0.14			
S-Magnesium	Mg	0.14			
S-Prostate-specific antigen	PSA	0.093			
S-Total complement complex	TCC	0.076			
Corticosteroid therapy	Co	0.066			
P-Renin	Ren	0.056			
S-α1-antitrypsin	AT	0.032			
S-Complement factor 4	C4	0.025			
S-NT pro-brain natriuretic peptide	pBNP	0.015			
P-Factor IX	IX	0.0051			

Abbreviations: S  =  serum, P  =  blood plasma, B  =  blood.

a The relative importance of each variable to the 139-variable model was determined using the “Variable importance” module in the SIMCA software.

b Patients were classified as infected or non-infected by an independent infectious disease specialist blinded to the results of the biochemical analyses

### Removal of variables improves the quality of the infection model

We next discarded variables with low explanatory power, those with VIP values <1.0 (**Table 3**), and produced a new O-PLS model based on 55 variables. This model had similar explanatory capacity (R^2^Y = 0.50) as the model based on all 139 variables (R^2^Y = 0.47), i.e., was equally able to distinguish infected from non-infected febrile neutropenic patients (**Figure 2A** *vs* **2B**). Further, the 55-variable model was more stable than the original 139-variable model, as reflected by improved cross-validation (Q^2^Y = 0.32 as compared to −0.0082).

The variables that contributed most to the 55-variable model are shown in **Figure 2C**. Variables that were higher in the infected than in the non-infected patients have values above zero; the height of the columns reflects how much each variable contributes to the model. Variables that were more elevated in the infected compared to the non-infected patients included procalcitonin, prothrombin time (INR), bilirubin (total and conjugated  =  Bil and BilC), cortisol, IL-8 and IL-10, mean corpuscular hemoglobin concentration (MCHC), ferritin and prohepcidin (**Figure 2C**). Conversely, non-infected patients had higher white blood cell counts, including reticulocytes, lymphocytes, neutrophils, eosinophils and monocytes, and higher levels of the coagulation factors XII and X (columns pointing in the opposite direction to the infection variable), **Figure 2C**. Erythrocyte volume fraction (EVF), low-density lipoprotein (LDL), total iron-binding capacity (TIBC), cholesterol, hemopexin, transferrin, hemoglobin, and sodium were also higher in the non-infected patients than in those with sterile inflammation (**Figure 2C**). Being a smoker was negatively associated with having a microbiologically defined infection (**Figure 2C**).

Our aim was to develop a diagnostic method that could be used in a clinical setting. Thus, it was important to remove time-consuming analyses not performed on a daily basis in tertiary care hospitals. For example, white blood cell differential counts cannot be performed by automated procedures when cell counts are very low, as in severely neutropenic patients. Consequently, variables based on differential counts were discarded. However, certain non-routine analytes were kept, e.g. HMGB1 and cytokines, since they contributed strongly to the model and could easily be automated in the future. This pragmatic pruning procedure left us with a simplified model composed of 40 variables. This model had the same explanatory capacity (R^2^Y = 0.47) as the one based on 139 variables, and stability comparable to the model composed of 55 variables (Q^2^Y = 0.30). In essence, the same variables were shown to be associated with infection in the 40-variable as in the 55-variable model (**Figure 2D**).

### The infection model correctly classified 9/10 validation patients

A pitfall in multivariate modelling is that models based on data derived from one set of patients may not be applicable to a new set of patients, because of over-fitting of the model. It is therefore essential to test the performance of any model in a new group of patients. A validation set of patients (n = 10) was recruited using the same inclusion criteria as before, and categorized as having or not having a microbiologically defined infection in a blinded fashion by two infectious disease specialists, independent of one another. The capacity of the 40-variable model to correctly predict if each of the 10 validation set patients had a microbiologically defined infection or not was tested. The concordance between the model's predictions, the classifications made by the infectious disease specialists, and the results of the microbiological investigations are shown in **Table 4**. A prediction score of ≥0.5 indicated that the patient in question was infected according to the model. Four of the patients were judged to be infected by both of the infectious disease specialists, and by the model. A fifth patient (CW85) was categorized as infected by the model, but as non-infected by the two clinicians. This “discordant” patient had suspected septic arthritis and erysipelas of the left ankle. PCR analysis of joint fluid revealed the bacterium *Kocuria*. Finally, five patients were classified as non-infected by the model and the two clinicians.

**Table 4:** Prediction of infection in the validation set of patients.

Patient ID	Prediction score^a^	InfectiousDisease^b^	Microbe
CW86	0.87	Sepsis	*Escherichia coli*
CW84	0.83	Sepsis	Coagulase-negative staphylococci
CW82	0.68	Lung infection	*Aspergillus fumigatus*
CW83	0.65	Sepsis	*Pseudomonas aeruginosa* and Coagulase-negative staphylococci
CW85	0.63	None	*Kocuria* sp. in joint fluid (DNA)
CW90	0.48	None	
CW88	0.41	None	
CW80	0.33	None	
CW87	0.23	None	
CW81	0.23	None	

a Classification according to 44-variable infection model. Prediction score >0.5  =  infected, <0.5 =  not infected.

b Classification made by the infectious disease specialist.

Based on the clinical assessment of the infectious disease specialists, taken to be the “gold standard”, the model correctly categorized all of the patients with microbiologically documented infection (4/4) as infected, and 5/6 of the non-infected patients as non-infected, yielding a specificity of 83%, sensitivity of 100%, a positive predictive value of 0.80, and a negative predictive value of 1.0. We also calculated the positive and negative likelihood ratios of the model: LR+ (Sensitivity/(100-Specificity)  = 5.9 and LR- (100-Sensitivity)/Specificity  = 0.

## Discussion

Here we tested the hypothesis that it would be possible to discriminate microbial from sterile systemic inflammation via the identification of distinct patterns of biochemical and clinical parameters using pattern recognition modeling. Hematology patients undergoing chemotherapy have a greater or lesser degree of cell lysis. Infection in such patients, thus, has to be identified against a background of cell damage-driven inflammation. Accordingly, both the infected and the non-infected patients had reduced plasma levels of citrulline, an amino acid produced by enterocytes, whose levels are reduced upon damage to the intestinal mucosa [20], [21].

We achieved a clinically applicable model that could separate infected from non-infected febrile neutropenic hematology patients. Starting from a model based on 139 biochemical and clinical parameters, we finished with a model composed of 40 variables with maintained discriminatory capacity. This model was tested using 10 newly recruited febrile patients. The results of this validation showed that each of the four patients deemed to have a microbiologically verified infection by the two independent clinicians were also classified as being infected by the model. Five patients were classified as non-infected both by the clinicians and by the model. There was one discordant classification - a patient predicted by the model to be infected, but not by either of the clinicians who did not attach importance to the finding of *Kocuria* DNA in joint fluid. However, this gram-positive skin bacterium can cause opportunistic infections in hematology patients [22]. It is more essential to rule out an infection in a febrile hematology patient who can proceed with potentially curative chemotherapy, than it is to avoid treating an extra patient with antibiotics.

**Figure 3:**
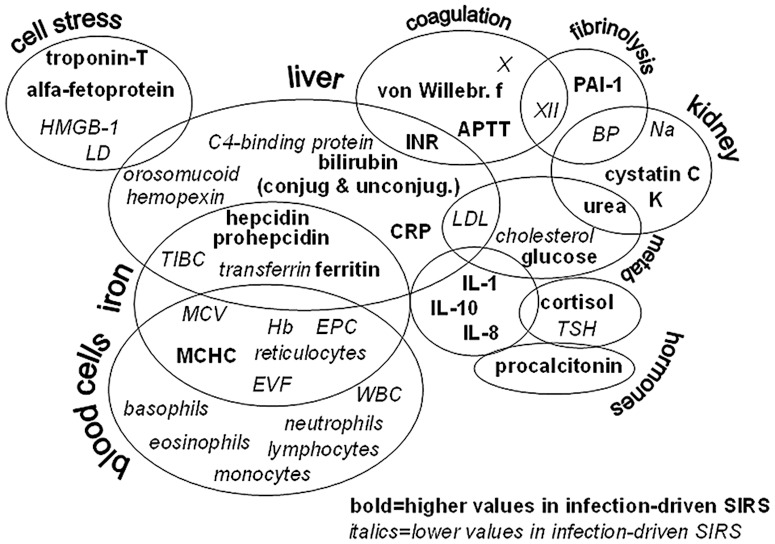
Key variables for segregation of systemic inflammation of infectious from non-infectious origin in neutropenic hematology patients presenting with fever. Variables are grouped based on tissue origin and function. BP  =  diastolic blood pressure, LD  =  lactate dehydrogenase, SIRS  =  systemic inflammatory reaction syndrome, TIBC  =  total iron-binding capacity, TSH  =  thyroid-stimulating hormone, X and XII  =  coagulation factors.

**Figure 3** maps the 40 variables that were discriminatory in our model. These biomarkers reflect the function of several biological pathways and organ systems, including coagulation, iron turn-over, bone marrow function and measures of organ and cell stress, underscoring the global nature of systemic inflammation. The strongest parameter was procalcitonin, which was more elevated in infected than non-infected patients. However, on its own, it could not be used to diagnose infection. In fact, a recent review of 3370 studies encompassing 178 putative sepsis biomarkers concluded that no single one of them could be used to diagnose sepsis [11].

One theory holds that the coagulation system arose chiefly to trap microbes [23]. In agreement with this hypothesis, the infected patients had lower levels of factors XII and X, prolonged coagulation time, and increased levels of plasminogen-activator inhibitor-1 [24] and von Willebrand factor [25] compared to patients with sterile inflammation. Conversely, protein S [26], an inhibitor of coagulation, was more elevated in sterile than infectious inflammation.

Iron is nearly mandatory for microbial growth and withholding iron is an important strategy of the body to starve them [27], [28]. Indeed, the infected patients had, as compared to the non-infected ones, raised levels of hepcidin (the chief negative iron regulator) [29], ferritin (which sequesters iron in the tissues), and reduced levels of transferrin (the iron transporting protein). In response to these adjustments, production of hemoglobin and red blood cells is depressed, which was reflected by more reduced hemoglobin levels and fewer erythrocytes and reticulocytes in infected than non-infected patients. Thus, even though anemia is a cardinal sign of all patients with severe hematological diseases, infected patients were more anemic than non-infected ones.

Cytokines are the prototype messenger molecules in inflammation. IL-8, IL-10 and IL-1 were more elevated in the infected than in the non-infected patients. IL-1β is a prototype pro-inflammatory cytokine with broad effects on metabolism, endothelial function and coagulation [30]. IL-8 is a neutrophil attractant, while IL-10 counteracts T cell activation and promotes antibody production [31]. IL-6 could not differentiate between sterile and microbe-driven inflammation, as it was similarly raised in both conditions. Acute phase proteins are produced by hepatocytes in response to a mixture of inflammatory mediators, e.g. IL-1, TNF, IL-6. C-reactive protein was elevated over baseline in all patients, but subtly more so in those with verified infection. Hence, CRP was included among the 40 variables in our model. Hemopexin, orosomucoid, and C4-binding protein also contributed to the model as they were instead more elevated in patients with sterile inflammation. Upon cell lysis, hemoglobin and heme groups are released [32]. These toxic compounds are rapidly cleared via the concerted action of haptoglobin, hemopexin and orosomucoid [33], [34]. Several acute phase reactants were similarly elevated in all patients, these included serum amyloid factor A, fibrinogen, α1-antitrypsin, ceruloplasmin, α2-macroglobulin and haptoglobin.

Since neutropenia was an inclusion criterion, all study patients had white blood cell counts below the normal range. This deficit was even more aggravated in the patients with proven infection, and occurred across all species of white blood cells. Leukocytes are depleted from the bloodstream by recruitment into infectious foci and adherence to endothelial cells activated by inflammation. An alternative explanation for the extra low white blood cell counts in infected neutropenic patients is the well-known fact that the severity of neutropenia is correlated to the risk of infection [4].

Metabolic alterations are a hallmark of systemic inflammation. Diminished levels of cholesterol and cholesterol-rich low-density lipoprotein, LDL, characterized all inflamed neutropenic patients, but especially those with proven infection. Cholesterol is a substrate for the synthesis of cortisol; the greater reduction of cholesterol and LDL in the infected patients fits with their relatively more elevated serum cortisol levels. Depressed serum sodium levels were even more pronounced in the infected patients. Hyponatremia develops when antidiuretic hormone (vasopressin) is released, presumably to counteract the low blood pressure that characterizes severe inflammation [35]. The infected patients did have lower diastolic blood pressure than the non-infected ones.

Another characteristic of systemic inflammation is organ stress. The infected patients, in contrast to those with sterile inflammation, had modestly raised serum cystatin C and troponin T levels, reflecting kidney and heart muscle stress, respectively. Troponin T is raised in sepsis [36]. Infected patients also had relatively higher serum levels of total and conjugated bilirubin and of alpha-fetoprotein. Bilirubin is a break-down product of heme whose biliary excretion is counteracted by intrahepatically produced IL-1 and IL-6 in sepsis [37], while alpha-fetoprotein is produced in the regenerating liver following hepatitis or intoxication [38], [39]. Collectively, these markers indicate a greater degree of liver stress in the infected patients. HMGB1, on the other hand, was associated with non-infectious systemic inflammation. This protein can be released both by injured host cells or immune cells exposed to microbes [40].

Our model must be regarded as a prototype that needs further refinement and validation considering the moderate number of patients used to construct the model and to validate the same model. The estimates of the model's specificity and sensitivity should also be interpreted with caution. However, if 30-40 biochemical analyses can be performed within a few hours at a reasonable cost, this pattern recognition-based strategy may revolutionize how we diagnose fever of unknown origin in the near future. Hopefully, this may improve clinical decision-making, leading to decreased morbidity and mortality in this vulnerable group of patients.
